# Calreticulin Expression Controls Cellular Redox, Stemness, and Radiosensitivity to Function as a Novel Adjuvant for Radiotherapy in Neuroblastoma

**DOI:** 10.1155/2023/8753309

**Published:** 2023-01-06

**Authors:** Chih-Jung Chen, Ya-Chuan Hu, Yueh Chien, Wei-Chieh Huang, Chi-Sheng Wu, Chung-Ying Tsai, Yang-Hsiang Lin, Meng-Shiue Lee, Chian-Shiu Chien, Yi-Ping Yang, Meng-Chou Lee, Chung-Chih Tseng, Hsiang-Cheng Chi

**Affiliations:** ^1^Department of Pathology and Laboratory Medicine, Taichung Veterans General Hospital, Taichung, Taiwan; ^2^School of Medicine, Chung Shan Medical University, Taichung, Taiwan; ^3^Department of Post-Baccalaureate Medicine, College of Medicine, National Chung Hsing University, Taichung, Taiwan; ^4^Nutrition Room of Zuoying Branch of Kaohsiung Armed Forces General Hospital, Kaohsiung City, Taiwan; ^5^Department of Medical Research, Taipei Veterans General Hospital, Taipei, Taiwan; ^6^College of Medicine, National Yang Ming Chiao Tung University, Yangming Campus, Taipei, Taiwan; ^7^Graduate Institute of Integrated Medicine, China Medical University, Taichung, Taiwan; ^8^Chinese Medicine Research Center, China Medical University, Taichung, Taiwan; ^9^Molecular Medicine Research Center, Chang Gung University, Taoyuan, Taiwan; ^10^Department of Otolaryngology-Head & Neck Surgery, Chang Gung Memorial Hospital, Taoyuan, Taiwan; ^11^Kidney Research Center, Department of Nephrology, Chang Gung Memorial Hospital, Taoyuan, Taiwan; ^12^Liver Research Center, Chang Gung Memorial Hospital, Linkou, Taoyuan, Taiwan; ^13^Department of Aquaculture, National Taiwan Ocean University, Keelung City, Taiwan; ^14^Center of Excellence for Ocean Engineering, National Taiwan Ocean University, Keelung City, Taiwan; ^15^Center of Excellence for the Oceans, National Taiwan Ocean University, Keelung City 20224, Taiwan; ^16^Institute of Medical Science and Technology, National Sun Yat-Sen University, Kaohsiung City, Taiwan; ^17^Zuoying Branch of Kaohsiung Armed Forces General Hospital, Kaohsiung City, Taiwan

## Abstract

Radiotherapy (RT) is currently only used in children with high-risk neuroblastoma (NB) due to concerns of long-term side effects as well as lack of effective adjuvant. Calreticulin (CALR) has served distinct physiological roles in cancer malignancies; nonetheless, impact of radiation on chaperones and molecular roles they play remains largely unknown. In present study, we systemically analyzed correlation between CALR and NB cells of different malignancies to investigate potential role of CALR in mediating radioresistance of NB. Our data revealed that more malignant NB cells are correlated to lower CALR expression, greater radioresistance, and elevated stemness as indicated by colony- and neurospheroid-forming abilities and vice versa. Of note, manipulating CALR expression in NB cells of varying endogenous CALR expression manifested changes in not only stemness but also radioresistant properties of those NB cells. Further, CALR overexpression resulted in greatly enhanced ROS and led to increased secretion of proinflammatory cytokines. Importantly, growth of NB tumors was significantly hampered by CALR overexpression and was synergistically ablated when RT was also administered. Collectively, our current study unraveled a new notion of utilizing CALR expression in malignant NB to diminish cancer stemness and mitigate radioresistance to achieve favorable therapeutic outcome for NB.

## 1. Introduction

In the past two decades, radiotherapy (RT) has been a major cost-effective cancer treatment applied to more than half of cancer patients worldwide [1]. In fact, about 60% of patients who show better prognostic outcome received RT as part of their treatment [2], while nearly 40% of cancer cures included RT that was singly administrated or in combination with other modalities [3]. RT has been an essential part of many multimodal treatments in clinics and provided enormous benefits to various cancer patients; nonetheless, effectiveness of RT has also been hampered by acquired chemoresistance and radioresistance [4]. Patients who suffer cancer relapse are often attributed to tumors that have metastasized or developed resistance to RT or chemotherapy treatments administered.

Therapeutic effects of RT arise from ionizing photons of X-ray, which generates reactive oxygen species (ROS) and causes DNA double strand breaks that lead to severe damage in tumor tissues. In the past decade, transcriptomic, genomic, and proteomic multidisciplinary approaches have been undertaken in order to identify new prognostic or predictive biomarkers and targets suitable for therapeutic intervention against cancer resistance [5, 6]. To broaden the therapeutic window of radiotherapy, novel biomarker-orientated treatments are suggested to be the next era of precision medicine in radiation oncology [7]. Enormous effects have been made towards discovery of radiosensitizers in aim to conquer undesirable radioresistance. Recently, increasing number of studies have demonstrated that in addition to conventional mechanisms of ROS and DNA damage repair (DDR), numbers of signaling molecules that regulate stress, hypoxic, and immune responses within tumor microenvironment are also responsible for the development of radioresistance, eventually leading to cancer recurrence and metastasis [8–12]. Elevated levels of molecular chaperones such as heat shock proteins (HSPs) in tumors that developed chemoresistance, for instance, are reported to promote tumor cell survival by mediating cytoprotection against radiation-induced cell death [13]. Such chaperone-targeting strategy by an HSP-immunogenic peptide was utilized to eradicate radioresistant subpopulations by enhanced CD4, CD8, and effector T cells in mammary tumors [14]. Interestingly, another study also demonstrated that inhibition of HSP90 could result in radiosensitization to radioresistant sarcoma cells as evidenced by delayed *γ*-H2AX clearance [15]. To date, very little is known about the role of chaperone CALR in radiation oncology nor any CALR-facilitated RT modality has been reported.

NB is a lethal pediatric tumor originated from primitive neural crest precursors of adrenosympathetic lineage and is frequently diagnosed during early childhood [16, 17]. Despite mounting efforts from more than 30,000 articles on neuroblastoma, this devastating pediatric cancer remains an ever-improving task for clinics and pharmaceutical industries. Current modalities for NB patients include chemotherapy, RT, and differentiating agents such as retinoids [18]. Regardless of recent success in immunotherapy and advanced RT such as proton beam therapy against NB [19], event-free survival rate for high-risk patients remains less than 50% as many patients initially respond to modalities but relapse with metastatic and resistant diseases [20, 21]. Hence, there has been an urgent need to competently improve therapeutic efficacy from current RT, preferably preventing the development of cellular resistance.

Similar to various HSPs, endoplasmic reticulum chaperone CALR is known for its ability in activating anticancer immunity by immunogenic cell death (ICD) [22], and expression levels of CALR have been utilized for novel therapeutic strategies via elevation of anticancer immunity in various cancer types including colorectal carcinoma, non-small-cell lung carcinoma, acute myeloid leukemia, and ovarian cancer [23–25]. The successful development of anti-CALR antibody for detection of ICD has greatly facilitated early assessments of therapeutic efficacies [26]. In present study, we thus investigated the underrated role of CALR in NB by unprecedentedly uncovering CALR-mediated regulation of radioresistance and cancer stemness.

## 2. Materials and Methods

### 2.1. Cells and Reagents

Neuroblastoma cell line SK-N-BE2C, SK-N-SH, and SH-SY5Y were purchased from American Type Culture Collection (ATCC, Manassas, VA, USA) and were maintained in 1 : 1 ratio of Eagle's modified Eagle's medium/F12 (EMEM/F12) supplemented with 10% fetal bovine serum (FBS) and cultured at 5% CO_2_ incubator. The following reagents and primary antibodies were used at the manufactures recommended dilutions: blocking solution (TFU-BL500, BIOTOOLS Co., Ltd., Taipei, Taiwan), enhanced chemiluminescent solution (TU-ECL02, BIOTOOLS Co., Ltd., Taipei, Taiwan), prestain protein marker (TM-PM10170-10, BIOTOOLS Co., Ltd., Taipei, Taiwan), and stripping buffer (TW-ST500, BIOTOOLS Co., Ltd., Taipei, Taiwan). Antibody against anti-CALR (ab2903) was purchased from Abcam (Cambridge, MA, USA), anti-NF-*κ*B p65 (D14E12) and I-*κ*B (44D4) from Cell Signaling Technology, anti-*β*-actin (A5441) from Sigma (St. Louis, MO, USA), and anti-H1 (H-2, sc-393358) from Santa Cruz Biotechnology (Dallas, TX, USA). siRNAs that specifically target CALR (AM16708) were purchased from Thermo Fisher (Waltham, MA, USA).

### 2.2. Irradiation

NB cells were irradiated at escalating doses of 0, 2, 4, 6, or 8 Gy (600 MU/min dose rate) at room temperature using X-ray unit (MBR-1505R; Hitachi, Hitachi, Japan) or a linear accelerator (RapidArc®, Varian, Palo Alto, USA). Dosages delivered were measured using radiation monitor.

### 2.3. Overexpression and Knockdown of CALR

Overexpression of CALR was performed by transfecting NB cells with a pcDNA3.1 plasmid that contains full-length *CALR* cDNA using Lipofectamine 2000 (Life Technologies, Camarillo, CA, USA). Stable cell NB lines overexpressing CALR variants were generated by lentiviral construct pLKO-AS2-*CALR*. Short hairpin (sh)RNA lentiviral vectors that are specifically targeting human *CALR* were purchased from the National RNAi Core Facility (Academia Sinica, Taipei, Taiwan). HEK293T cells were utilized for preparation of lentiviral particles containing sh*CALR*. Lentiviral transduction was carried out with tittered lentiviral particles for 24 hours before puromycin selection initiated for selection of successfully transduced NB cells. Silencing of *CALR* using siRNA specifically against *CALR* (si*CALR*, Santa Cruz, sc-29234) was conducted using RNAiMAX transfection reagent (Thermo Fisher, 13778150) according to manufacturer's instruction.

### 2.4. Neurospheroid Assay

Neurospheroid cultures were carried out in conditional medium with marginal modification as previously reported [27]. Briefly, NB cell lines were cultured and used to form neurospheroids at 20,000 cells per 24-well (ultralow attachment) for three days prior to first morphological assessments. The medium was then replenished every 3 days and subcultured when spheres reached over 150 *μ*m in diameter to avoid clogs. Conditional medium applied contained bFGF, EGF, insulin, and StemPro® Neural Supplement (Thermo Fisher Scientific) that were added fresh before use.

### 2.5. Immunoblot

NB cell lysates were isolated using RIPA buffer (Sigma, St. Louis, MO, USA). Protein concentrations of cleared lysates were determined with a BCA protein assay reagent kit (Pierce, Rockford, IL, USA). Equivalent amounts of proteins as determined by BCA protein assay were denatured before resolving in polyacrylamide gels for SDS-PAGE and chemiluminescent HRP detection (Millipore, Billerica, MA, USA) using Gel Doc Imager (Bio-Rad, Hercules, CA, USA).

### 2.6. Quantitative Real-Time PCR (qPCR)

Total RNA was extracted from neuroblastoma cells with TOOLSmart RNA Extractor (DPT-BD24, BIOTOOLS Co., Ltd., Taipei, Taiwan). qPCR was conducted according to manufacturer's instruction from ToolsQuant II Fast RT kit (KRT-BA06, BIOTOOLS Co., Ltd., Taipei, Taiwan) using primers listed in Supplementary Table S1, and 1 × TOOLS SuperFast SYBR Green qPCR mix (FPT-BB07, BIOTOOLS Co., Ltd., Taipei, Taiwan).

### 2.7. NB Xenograft Model

NB xenograft model was established using SK-N-BE2C cells. Immunodeficient nod/scid mice were transimplanted with viable SK-N-BE2C cells at 1 x 10^6^ treated with or without lentiviral particles overexpressing CALR in PBS/matrigel to left thighs of mice that were randomly grouped. RT was delivered twice per week for 3 weeks after transimplantation of cells. All growth of xenografts were allowed to progress until the largest tumor volume reached 1500 mm^3^ to meet institutional animal ethic guidelines. The two longest perpendicular axes in the x/y plane of each xenograft tumor were recorded by using calipers as tumor volumes were calculated according to equation: xenograft volume = xy^2^/2, as standard practice [28]. Statistical significances in tumor growth between different groups were determined using linear regression analysis from Prism 9. All animal studies were approved by the Institutional Animal Care and Use Committee of the Chang Gung University, Taiwan.

### 2.8. Flow Cytometry and Cellular ROS Detection

Expression of cellular surface CD133 and cellular ROS level was determined using flow cytometry. 0.05 *μ*g of FITC-conjugated CD133 antibody (EMK08, Thermo Fisher Scientific) was used for each reaction containing 10^6^ cells. Total ROS in cells treated with CALR-overexpressing lentiviral particles were determined using 1X ROS assay stain of the ROS Assay Kit (88-5930, Thermo Fisher Scientific) according to manufacturer's instructions.

### 2.9. Statistical Analyses

All statistical analyses in this study were conducted using one-way ANOVA unless otherwise stated.

## 3. Results and Discussion

### 3.1. CARL Expression Is Closely Correlated to Neuroblastoma Malignancy under Radiotherapy

**Figure 1:**
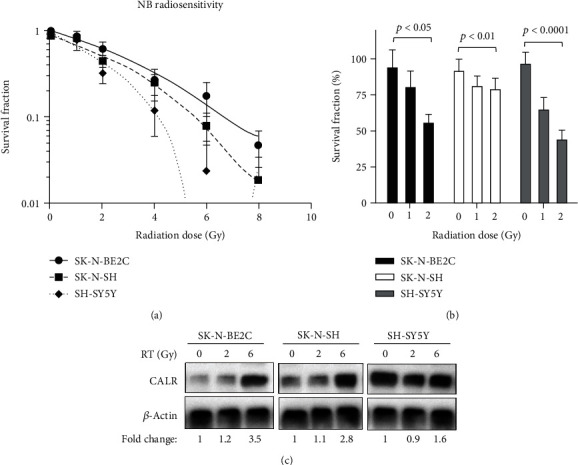
CALR expression is irradiation-inducible in three commonly studied human neuroblastoma cell lines. (a) Radiosensitivity was determined by measuring survival fraction of SK-N-BE2C, SK-N-SH, and SH-SY5Y cells that were exposed to escalating doses of radiation at 0, 2, 4, 6, and 8 Gy. (b) SF2 of each cell line was calculated by measuring cells survived at 0, 1, and 2 Gy. (c) Three NB cell lines were exposed to IR at 0, 2, or 6 Gy and protein expression of CALR was assessed and quantitatively normalized to expression of *β*-actin.

Although increasing body of evidence has suggested the importance of CSCs in mediating cellular radioresistance for different solid tumors, information for biological significance of CSC in NB malignancy remains limited. Since intrinsic radiosensitivity of cancer cells or tumors has been routinely determined based on their colony-forming abilities [29, 30], our pilot study first established radiosensitivity profiles for three of the most widely studied neuroblastoma cell lines, including SK-N-BE2C, SK-N-SH, and SH-SY5Y. In Figure 1(a), we studied survival fraction of these cell lines by exposing to escalating doses of ionizing radiation (IR) at 0, 2, 4, 6, and 8 Gy. Survival fraction analysis indicated that SH-SY5Y was most radiosensitive to ionizing radiation at the range between 0 to 8 Gy when compared to the more malignant SK-N-SH or SK-N-BE2C cells. The most malignant SK-N-BE2C cells appeared to be most radioresistant to IR. To substantiate radiosensitivity of these NB cells for further studies, survival fraction at 2 Gy (SF2) was ascertained. The results demonstrated greater radiosensitivity in the order of SH-SY5Y, SK-N-SH, and SK-N-BE2C by comparing unirradiated cells to cells treated with 2 Gy of IR (*p* < 0.0001, *p* < 0.01, and *p* < 0.05, respectively, Figure 1(b)), affirming SH-SY5Y as most radiosensitive NB cells.

Strategic immunotherapies that involved CALR-mediated activation of immune responses to treating multiple cancers for have been documented [26, 31, 32]; nonetheless, whether CALR plays functional role in radiotherapy is yet to be delineated. In present study, we explored whether CALR could be impacted by irradiation or mediate cancer malignancies of NB. Our data demonstrated that protein expression of CALR was markedly upregulated in all three NB cell lines when exposed to RT (Figure 1(c)). Interestingly, protein expression levels of CALR correlated in proportion to radiosensitivity observed in Figures 1(a) and 1(b) as most radiosensitive SH-SY5Y cells expressed highest level of CALR when treated with 6 Gy of IR. Of note, CALR expression was significantly augmented in the most malignant and radioresistant SK-N-BE2C cells that received 6 Gy of IR (Figure 1(c)), implicating a potential of CALR in the regulation NB radioresistance.

### 3.2. Neurospheroid Formation Is Mitigated in NB Cells when CALR Expression Is Induced by RT

**Figure 2:**
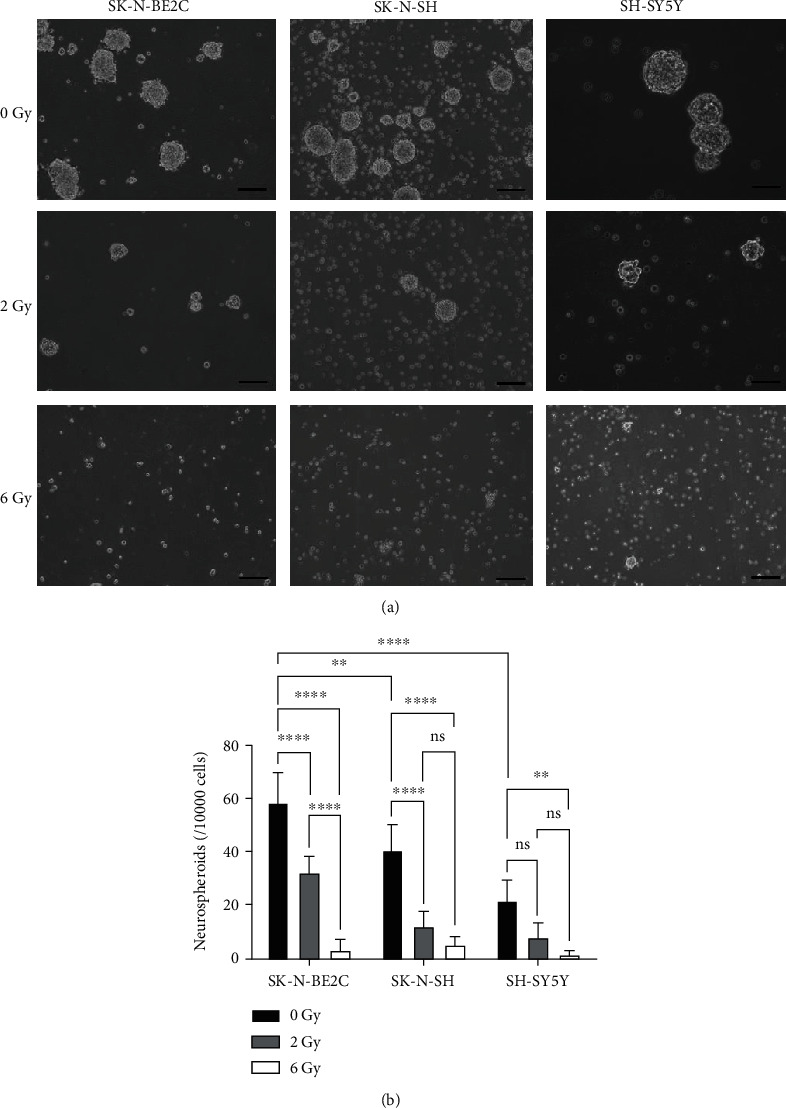
NBCSCs (neurospheroids) cultured from NB cell lines elicit differential levels of radioresistance to RT. (a) Neurospheroids cultured from three NB cell lines, SK-N-BE2C, SK-N-SH, and SH-SY5Y were cultured as described in Materials and Methods and treated with RT at 0, 2, or 6 Gy. (b) Resulting neurospheroids were counted and quantitatively analyzed by one-way ANOVA (^∗∗^*p* < 0.01, ^∗∗∗^*p* < 0.001, ^∗∗∗∗^*p* < 0.0001, and ns: nonsignificant).

CSCs are small-cell populations reported to be highly resistant to conventional therapies such as chemotherapy and radiation in treating cancerous tumors including NB [33, 34]. To corroborate the correlation between CALR expression and formation of neuroblastoma cancer stem cells (NBCSCs) in the context of RT, we firstly scrutinized neurospheroid-forming ability of all three NB cell lines. Our results showed consistent observations made in Figure 1 as SK-N-BE2C was able to form significantly more neurospheroids than SK-N-SH and SH-SY5Y (*p* < 0.01 and *p* < 0.0001, respectively, Figures 2(a) and 2(b)). When treated with 2 Gy of RT, the numbers of neurospheroid formed from both SK-N-BE2C and SK-N-SH were significantly lowered when compared to unirradiated cells (both *p* < 0.0001). At 6 Gy of RT that greatly induced CALR protein expression (Figure 1(c)), neurospheroids formed from all NB cells were considerably eliminated (Figure 2), implicating an critical role of CALR in modulating radioresistance of NB cells.

### 3.3. Silencing CALR Renders NB Cells more Stem-Like and Resistant to RT

**Figure 3:**
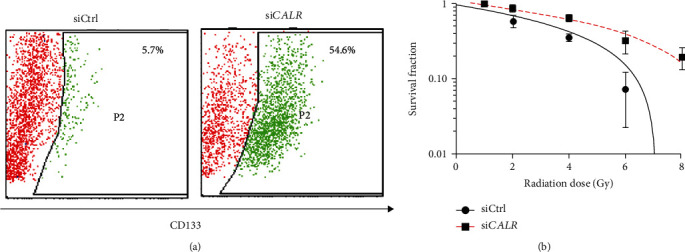
Effect of *CALR* expression on NB stemness and survival fraction to escalating radiation in SH-SY5Y cells. (a) SH-SY5Y cells were transfected with siRNA control (siCtrl) or siRNA specifically against *CALR* (si*CALR*) and subjected to analysis of CD133 population by flow cytometry. (b) si*CALR*-transfected SH-SY5Y cells were subsequently subjected to RT at 0, 2, 4, 6, or 8 Gy to examine differences in survival fraction by comparison to siCtrl-transfected cells.

To ascertain whether CALR indeed is capable of mediating NB radioresistance, we silenced *CALR* expression in endogenously CALR high-expressing SH-SY5Y cells and examined for influences on cancer stemness and survival fraction. As shown in Figure 3(a), cells that were depleted of *CALR* showed greatly increased NBCSC population as indicated by CD133. In addition, si*CALR* cells demonstrated greater survival fraction when compared to control siRNA- (siCtrl-) transfected cells in clonogenic assays (Figure 3(b)), indicating the importance of lowering *CALR* in maintaining cellular resistance to RT. These results thus implicated a critical correlation of *CALR* expression to modulating radioresistance as silencing of *CALR* led to significantly increases in CD133^+^ NBCSCs that potentially contributed to enhanced RT resistance in *CALR-*depleted SH-SY5Y cells.

### 3.4. Manipulating CALR Expression Elicits Reciprocal Stemness Regulation in NB

**Figure 4:**
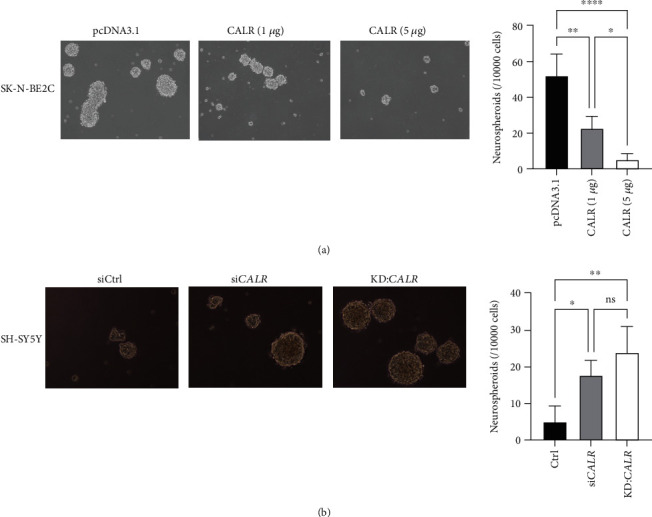
CALR overexpression and knockdown show reciprocal control over neurospheroid formation. (a) SK-N-BE2C cells were transfected with empty pcDNA3.1 vector, pcDNA3.1-CALR at 1 or 5 *μ*g, and were subsequently grown into neurospheroids in low-attachment 6-well plates. (b) SH-SY5Y cells were transfected with siCtrl or siCALR or infected with lentiviral shCALR; resulting cells were subjected to neurospheroid formation assays. Neurospheroids larger than 200 *μ*m were recorded and quantitatively analyzed using one-way ANOVA (^∗^*p* < 0.05, ^∗∗^*p* < 0.01, and ns: nonsignificant).

Following the above observation on the role of CARL in promoting NB stemness and elevating resistance in response to RT when *CALR* expression was silenced, we next investigated whether ectopic overexpression in CALR low-expressing SK-N-BE2C cells could impinge on NB stemness and radioresistance. Our results demonstrated that a dose-dependent effect of CALR overexpression was observed as 1 *μ*g of CALR overexpression in SK-N-BE2C cells led to significantly reduced number of neurospheroids formed (*p* < 0.01), while 5 *μ*g of CALR overexpression further abrogated neurospheroid formation when compared to pcDNA3.1 or CALR (1 *μ*g) (*p* < 0.0001 or *p* < 0.05, respectively, Figure 4(a)). Further, the impact of silencing *CALR* on NB stemness regulation was substantiated by establishing *CALR* stable knockdown (KD:*CALR*) cells. Our data showed that both transient si*CALR* and KD:*CALR* resulted in significantly lowered neurospheroid formation in CALR high-expressing SH-SY5Y cells (*p* < 0.05 and *p* < 0.01, respectively, Figure 4(b)).

### 3.5. Cancer Stemness Genes Are Effected by CALR in NB

**Figure 5:**
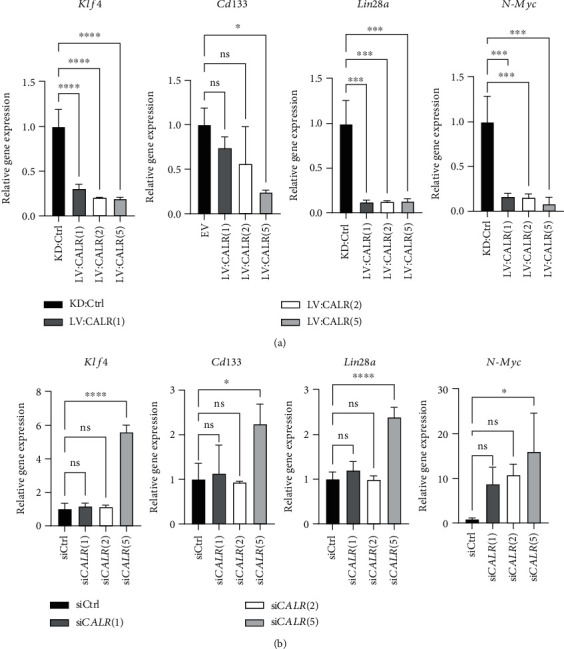
Expression level of CALR is closely correlated to regulation of NB stemness genes. (a) SK-N-BE2C cells that are CALR endogenous-low were infected with empty lentiviral particle (LV) carrying no targeting shRNA (KD:Ctrl) or CALR-expressing at MOI of 1, 2, or 5 (LV:CALR (1), LV:CALR (2), or LV:CALR (5)). After puromycin selection for 3 days, NB stemness gene expressions of *Klf4, CD133, Lin28a*, and *N-Myc* were quantitatively measured by qPCR and statistically analyzed (^∗^*p* < 0.05, ^∗∗∗^*p* < 0.001, and ^∗∗∗∗^*p* < 0.0001). (b) SH-SY5Y cells were transfected with siCtrl or siCALR (1, 2, or 5 *μ*g) and allowed to grow for 2 days prior to qPCR analyses on expression of the stemness gene. (^∗^*p* < 0.05, ^∗∗∗∗^*p* < 0.0001).

Our findings from Figures 3 and 4 that suggested a pivotal role of CALR in regulation of NB stemness and radioresistance prompted us to inspect CALR-mediated impact on expression of common stemness genes including *Klf4*, *CD133*, *Lin28a*, and *N-Myc* [35–38]. Our results revealed that *Klf4*, *CD133*, *Lin28a*, and *N-Myc* were all significantly downregulated when *CALR* was overexpressed in more malignant SK-N-BE2C cells (Figure 5(a)). In contrast, silencing *CALR* in less malignant SH-SY5Y cells conversely resulted in significantly elevated expression of *Klf4*, *CD133*, *Lin28a*, and *N-Myc* (Figure 5(b)). These results therefore suggested an important role of CALR in modulating stemness of NB cells.

### 3.6. CALR Overexpression Is Associated with ROS and Proinflammatory Levels in NB

**Figure 6:**
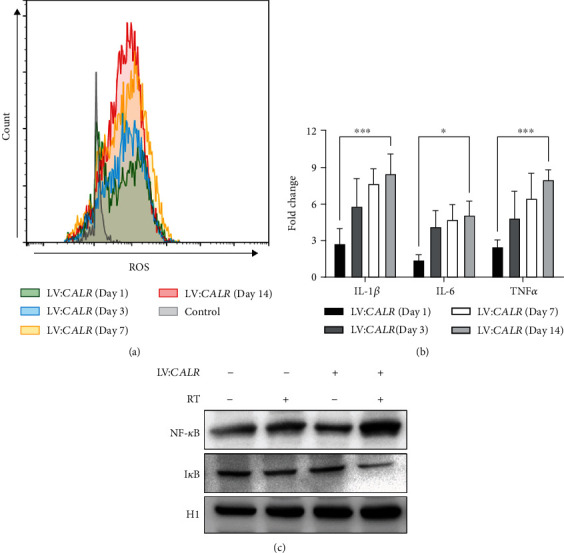
CALR overexpression is associated with cellular levels of ROS and proinflammatory molecules in SK-N-BE2C cells. (a) SK-N-BE2C cells were infected with lentiviral particles overexpressing CALR cDNA, and infected cells were selected with puromycin as described in Materials and Methods. Infected and selected cells were subjected to ROS detection by flow cytometry. (b) Cell culture media from CALR-overexpressing SK-N-BE2C cells were collected at day 1, 3, 7, and 14 and were used for detecting secretorylevels of IL-1*β*, IL-6, and TNF*α*. (c) Nuclear extracts of CALR-overexpressing SK-N-BE2C cells treated with RT were harvested and subjected to western blotting analysis on nuclear protein levels of NF-*κ*B and I*κ*B. Histone H1 was used as internal control for nuclear protein expression.

Although activity of molecular chaperones such as heat shock protein (HSP) family is known as redox switch that protects cells from oxidative stress [39], the role of endoplasmic reticulum chaperone CALR in mediating cellular redox still remains largely elusive. We thus next investigated whether manipulating *CALR* expression could also impinge on cellular redox in NB cells. By overexpressing *CALR* with lentivirus in SK-N-BE2C cells, we observed a systemic rise in cellular ROS levels after day 1, and the elevation persisted throughout the establishment of *CALR*-overexpressing cell line till day 14 (Figure 6(a)). Since cellular ROS reportedly enhances activities of mitogen-activated protein kinase (MAPK) as well as secretion of several interleukins and cytokines [40], we further analyzed secretion of interleukins IL-1*β*, IL-6, and tumor necrosis factor *α* (TNF*α*). As shown in Figure 6(b), our results showed consistent and significant increases in the secretion of IL-1*β*, IL-6, and TNF*α* when comparing day 14 to day 1 after *CALR* overexpression. Furthermore, expression of transcription factor nuclear factor kappa B (NF-*κ*B) that is frequently activated during oxidative stress in the context of RT was examined. Our data demonstrated that both *CALR* overexpression and RT led to markedly elevated nuclear translocation of NF-*κ*B, while RT in *CALR*-overexpressed cells showed further enhancement in nuclear NF-*κ*B (Figure 6(c)). Moreover, the inhibitory I*κ*B that associates with NF-*κ*B in the cytoplasm was greatly reduced in RT-treated *CALR*-overexpressing cells. Collectively, these results implicated that *CALR* overexpression could effective alleviate stemness and radioresistance via association with elevated cellular ROS and proinflammatory cytokines in NB.

### 3.7. CALR Overexpression Synergistically Reduces NB Tumors Treated with RT

**Figure 7:**
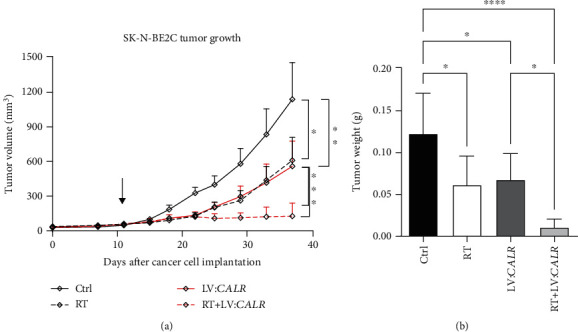
Ecotpoic CALR expression and RT synergistically alleviate tumor growth of NB. (a) SK-N-BE2C cells with or without LV:CALR infection were employed to establish NB xenograft model. RT (1 Gy) was delivered to all mice bearing xenografts that reached average of 50 mm^3^ twice weekly (black arrow) for 3 weeks before harvest of tumors for tumor weight measurements. (b) Statistical differences between the treatment groups for tumor growth or tumor weight were analyzed by linear regression and one-way ANOVA, respectively (^∗^*p* < 0.05, ^∗∗^*p* < 0.01, ^∗∗∗^*p* < 0.001, and ^∗∗∗∗^*p* < 0.0001).

To translationally substantiate aforementioned findings in *CALR*-mediated NB stemness, radioresistance, and cellular redox, we employed NB xenograft to investigate the individual and collective effects of *CALR* overexpression and RT on in vivo tumor growth of NB. The results revealed that the growth of NB xenografts from *CALR*-overexpressing cells or RT-treated cells was significantly reduced when compared to control cells (*p* < 0.05 and *p* < 0.01, respectively, Figure 7(a)). Importantly, RT-treated *CALR*-overexpressing xenografts showed synergistically reduced growth rate than that of *CALR*-overexpressing or control group (both *p* < 0.001). Xenografts were harvested and weighed for further statistical analysis that showed *CALR* overexpression and RT treatment not only synergistically decreased tumor weight when compared to the *CALR* -overexpression group (*p* < 0.05); the combined treatments elicited near ablation in tumor growth when compared to control group (*p* < 0.0001, Figure 7(b)). Moreover, limiting dilution analysis that is the gold standard method in cancer stemness determination [41] was employed to corroborate CALR-mediated stemness in vivo. As shown in Table 1, SK-N-BE2C cells were able to grow into tumor at a 0.02781% frequency, which was significantly higher than that of cells treated with RT or *CALR* overexpression (both *p* < 0.0001). Of note, combinatorial treatment of *CALR* overexpression and RT resulted in tumor formation frequency at 0.00007%, which was significantly lower than that of *CALR* overexpression or RT treatment alone (*p* < 0.05 and *p* < 0.01, respectively). Together, our data suggested that elevating the expression of *CALR* could greatly suppress NB tumor growth and act as an effective adjuvant regimen alongside RT.

## 4. Discussion and Conclusion

Low dose RT has been a well-established regimen in treating numbers of cancer types to minimize unwanted adverse cytotoxicity in normal tissues of radiosensitive cancer patients. Recently, accumulating efforts have been made from studies that investigated the molecular mechanisms or pathways responsible for radiotoxicity [42, 43]. A few latest studies by Toy et al., Pavlopoulou et al., and Mavragani et al. employ integrative bioinformatics strategies and unravel novel gene signatures as potential biomarkers accounting for radioresistance in a variety of cancers such as breast cancer, colon cancers, and leukemia [44–46]. Due to the lack of normal tissue availability in NB, unfortunately, potent genes responsible for NB radiosensitivity are yet to be identified. Data presented in current study thus unprecedentedly manifested CALR as a prominent gene that regulates radiosensitivity of NB. More importantly, since NB originated from primitive neural crest cell (NCC) precursors, we further analyzed whether CALR could impinge on radiosensitivity and stemness of NCCs derived from hPSCs. Our data demonstrated that neither survival fraction nor expression of stemness genes such as *Klf4*, *Lin28a*, *N-Myc*, and *Cd133* were influenced when *CALR* was silenced or overexpressed (Supplementary Figure 1), alleviating concerns of affecting normal tissues when CALR is utilized as RT adjuvant.

Aside from our current study that uncovered the function of CALR as therapy adjuvant for treating NB, surface CALR has been proposed as cancer treatment adjuvant for photodynamic therapy (PDT) [47]. Externally added recombinant CALR protein is shown to improve antitumor response elicited by PDT or PDT-generated vaccines in HNSCC tumor model. In pancreatic adenocarcinoma, higher CALR expression enhances antitumor immunity and is found to associate with improved survival rates [48], while CALR exerts immune responses against malignant high-grade serous carcinomas (HGSCs) albeit its prognostic value for HGSC patients remains unclear [32]. These antitumor activities elicited by ecto-CALR are attributed to its ability in activating phagocytic signal for ICD through dendritic cells and cytotoxic T-cells [49]. Despite the role of surface CALR responsible for ICD in several cancer types is well-documented, there has been a missing link between CALR and its function in cancer stemness regulation. In our present study, although surface translocation of CALR was not detected (data not shown), our data that revealed the activation of MAPK pathway via elevated nuclear NF-*κ*B expression and ROS-mediated proinflammatory pathway provided a new mechanism that underlined CALR-mediated stemness regulation in NB (Figures 5 and 6). Indeed, NF-*κ*B activity is recently suggested as a target of future drug development for neuroblastoma therapy due to its association with fenretinide-induced ROS to initiate apoptosis in SH-SY5Y cells [50].

Although overexpression of chaperones such as HSP47 is associated with ROS production [51], our observation in the secretions of IL-1*β*, IL-6, and TNF*α* induced by CALR-mediated ROS elevation in NB was unprecedented to date. Of note, our findings are also consistent with reported increases in cellular ROS that often lead to secretion of chemokines and proinflammatory cytokines via activations of key factors such as MAPK, extracellular signal-regulated kinases (ERKs), c-Jun N-terminal kinases (JNKs), and NF-*κ*B [40, 52]. In line with our observation in enhanced cancer stemness mediated by *CALR*-silencing in SH-SY5Y cells (Figures 4 and 5), exposure of SH-SY5Y cells to anti-inflammatory molecule 2,3,4′,5-tetrahydroxystilbene-2-O-*β*-D-glucoside (TSG) inhibited IL-6 and TNF*α* and prevented the cells from undergoing apoptosis [53]. With regards to neuronal disease such as Alzheimer's disease (AD), a recent study utilizes CALR-specific antibody to block neuronal CALR and shows induced oxidative neurotoxicity to prevent AD pathogenesis [54].

In addition to our significant findings in CALR-mediated ROS and proinflammatory cascade, to date, very little is known about whether CALR is involved in cellular resistance and stemness. A report that studied pancreatic CSCs (P-CSCs) show that CALR is highly expressed in P-CSCs and is associated with poorer survival outcome for pancreatic cancer patients [55]. Despite the lack of information in stemness regulation, CALR has been suggested as a prognostic marker that signifies tumor progression in cancer types of nonneuron origin [32, 56, 57]. For NB, in contrast, CALR has been long reported as a prognostic factor that favors better clinicopathological outcomes [58–60]. Therefore, our present study provided a founding discovery in the field of cancer stemness regulation mediated by CALR that could help strategize future therapy design in treating NB alongside RT. In aspect of radioresistance, moreover, activation in phosphorylation of ATM is known as the master regulator that contributes to radioresistance of CSCs via enhancing DNA repair efficiency [61, 62]. Consistently, our data further revealed significantly reduced phosphorylation of ATM as well as greatly impaired DNA damage clearance as indicated by *γ*H2A.X when NB cells were CALR-overexpressed and treated with RT (Supplementary Figure 2). In summary, our current study unraveled a new role of CALR in mediating stemness and radioresistance in NB and provided a new notion for future design in utilizing CALR as clinical RT therapy adjuvant for improving prognosis of NB patients.

## Figures and Tables

**Table 1:** ELDA for assessing impacts of CALR overexpression and RT on in vivo stemness of SK-N-BE2C xenograft model. Pairwise chi-square test was conducted to obtain statistical differences between each group (^∗^*p* < 0.05; ^∗∗^*p* < 0.01; ^∗∗∗∗^*p* < 0.0001).

Cell no. injected	1,000,000	100,000	10,000	1,000	Freq. (%)	*p* value
	Xenograft with tumor/total xenograft number					
Ctrl	5/5	5/5	4/5	3/5	0.02781	vs. ctrl+RT^∗∗∗∗^
Ctrl+RT	4/5	4/5	2/5	1/5	0.00054	vs. LV:*CALR*+RT^∗∗^
LV:*CALR*	3/5	3/5	2/5	1/5	0.00027	vs. ctrl^∗∗∗∗^
LV:*CALR*+RT	2/5	1/5	0/5	0/5	0.00007	vs. LV:*CALR*^∗^

## Data Availability

All data derived and analyzed during this study are included in this article. Data could be available upon request. Please send data requests to Dr. Hsiang-Cheng Chi, PhD. Graduate Institute of Integrated Medicine, China Medical University, Taichung, Taiwan.

## Supplementary Materials

Supplementary Materials Supplementary Figure 1: radiosensitivity and stemness of iPSC-derived neural crest cells (NCCs) that mimic sympathetic ganglion progenitor cells of NB are not influenced by CALR expression. Supplementary Figure 2: CALR overexpression results in radiosensitivity associated with impaired DNA damage repair abilities. Supplementary Table S1: qPCR primer direction and sequences.
