# Supplementary material for: Calreticulin Expression Controls Cellular Redox, Stemness, and Radiosensitivity to Function as a Novel Adjuvant for Radiotherapy in Neuroblastoma
Source: Oxid Med Cell Longev. 2023 Jan 6;2023:8753309. doi: 10.1155/2023/8753309 (PMC9839411; doi:10.1155/2023/8753309)
Supplement: Supplementary Materials — Supplementary Figure 1: radiosensitivity and stemness of iPSC-derived neural crest cells (NCCs) that mimic sympathetic ganglion progenitor cells of NB are not influenced by CALR expression. Supplementary Figure 2: CALR overexpression results in radiosensitivity associated with impaired DNA damage repair abilities. Supplementary Table S1: qPCR primer direction and sequences. [file 8753309.f1.docx]

**Supplementary Description**

**Supplementary Figure 1**

**
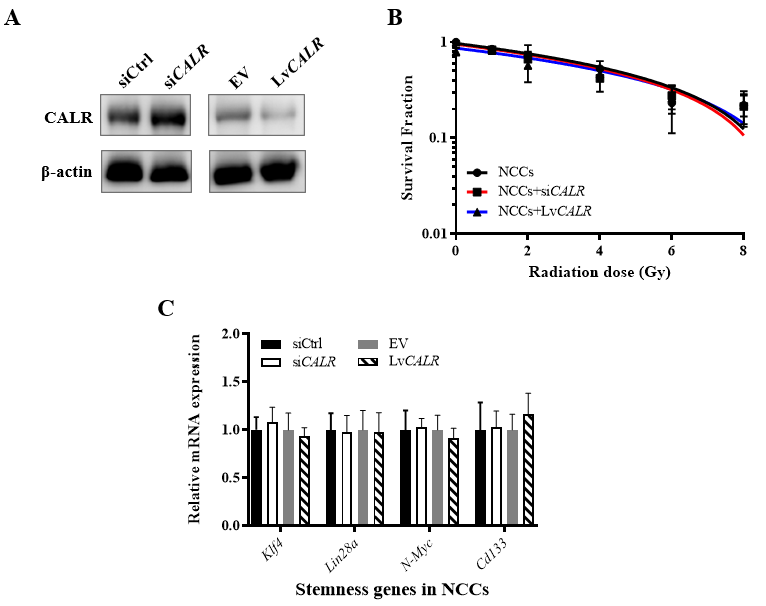
**

**Supplementary Figure 1. Radiosensitivity and stemness of iPSC-derived neural crest cells (NCCs) that mimic sympathetic ganglion progenitor cells of NB are not influenced by CALR expression.** (A) NCCs derived from WLS-1C cells were subjected to determination of CALR protein expression by western blotting prior to colony formation assay and survival fraction analysis (B). (C) Expression of stemness genes associated with NB malignancy investigated in current study including *Klf4*, *Lin28a*, *N-Myc* and *Cd133* in NCCs were examined by qPCR.

**Supplementary Figure 2**

**
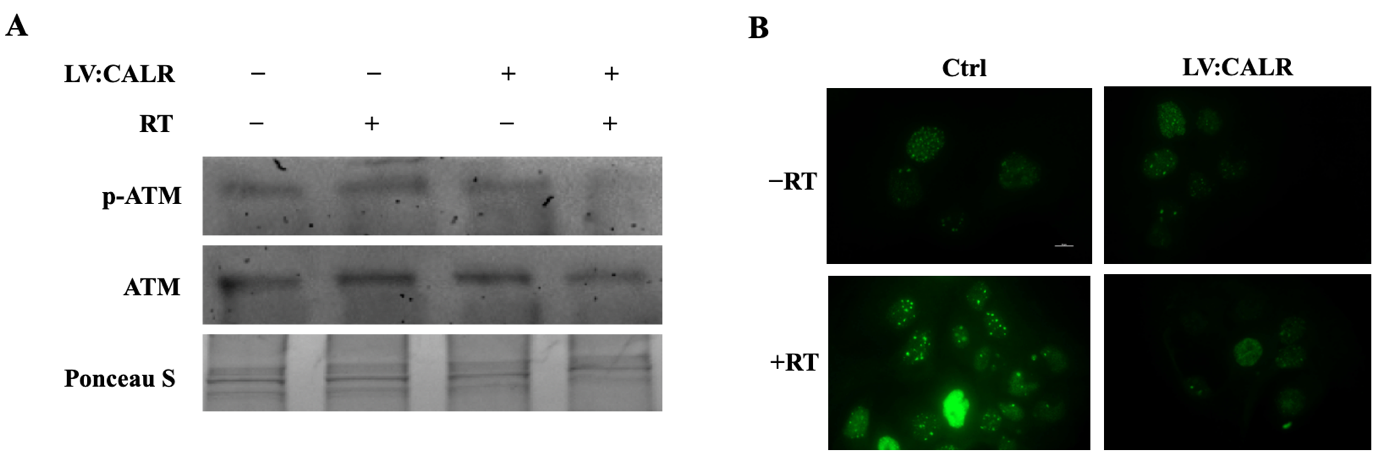
**

**Supplementary Figure 2. CALR overexpression results in radiosensitivity associated with impaired DNA damage repair abilities.** SK-N-BE2C cells overexpressed with CALR were treated with RT and subjected to analysis on the phosphorylation of ATM by western blotting (A) or levels of γH2A.X by immunofluorescence (B) 1 hour or 24 hours after RT, respectively.

**Supplementary Materials and Methods**

**Culture and differentiation of iPSC into NCCs**

Human iPSC line WLS-1C was purchased from Stem cell and maintained in mTeSR1 medium (StemCell Technologies) on cell culture plates coated with growth factor reduced matrigel matrix (Corning) and differentiated into neural crest cells (NCCs) as previously described [1, 2]. Briefly, WLS-1C cells were transfected with siRNAs or shRNA prior to cell aggregates were treated with CHIR99021 (2.0 μM) and cultured in Essential 6 Medium supplemented with RA (100 nM), BMP4 (50 ng/mL) and FGF2 (20 ng/mL) for 7 days. Resulting cell aggregates were dissociated and FACS-sorted (CD49d^+^eGFP^+^) and subjected to IR before culturing in Ultra-Low Attachment dishes or plates (Corning), from which cell colonies or total cell lysates were subjected to survival fraction or western blotting analysis, respectively.

**Construction of WLS-1C-eGFP cell line and flow cytometry analyses.**

The TALEN-based transfection of targeting Exon 3 of *PHOX2B* and selection of WLS-1C*^Phox2b::^*^eGFP^ cells were established as previously described [1]. Anti-eGFP (Abcam, ab6556) and anti-CD49D-PE (Abcam, ab28101) were used for validation of established cells according to manufacturer’s protocol on BD FACSAria II (BD Bioscience). Parental WLS-1C cells were used as negative controls for eGFP fluorescence.

**Supplementary Table S1**

**qPCR primer direction and sequences**

| **Gene** | **Direction** | **Sequence** |
| --- | --- | --- |
| *Klf4* | Forward | TTTCCTGCCAGACCAGAT |
| *Klf4* | Reverse | GTGTGCCTTGAGATGAGAAC |
| *Cd133* | Forward | AGGATGGATTCAGAGGATGT |
| *Cd133* | Reverse | CTGGGATTCTTTCCGAGTTAG |
| *Lin28a* | Forward | TTCGGCTTCCTGTCTATGA |
| *Lin28a* | Reverse | TCGCTCACTCCCAATACA |
| *N-Myc* | Forward | TGGTCACTAGTGTGTCTGT |
| *N-Myc* | Reverse | CTGCCTTGTTGTTAGAGGAG |
| *β-actin* | Forward | GGCTGTATTCCCCTCCATCG |
| *β-actin* | Reverse | CCAGTTGGTAACAATGCCATGT |

Primer sequences used for qPCR in experiments performed in Figure 5 and Supplementary Figure 1.

**Supplemental References**

1. Kirino, K., et al., *Efficient derivation of sympathetic neurons from human pluripotent stem cells with a defined condition.* Scientific Reports, 2018. **8**(1): p. 12865.

2. Okita, K., et al., *An efficient nonviral method to generate integration-free human-induced pluripotent stem cells from cord blood and peripheral blood cells.* Stem Cells, 2013. **31**(3): p. 458-66.
